# Mast Cells in Neurodegenerative Disease

**DOI:** 10.3389/fncel.2019.00171

**Published:** 2019-04-30

**Authors:** Michael K. Jones, Archana Nair, Mihir Gupta

**Affiliations:** ^1^Department of Medicine, Vascular Biology Center, Division of Hematology, Oncology and Transplantation, University of Minnesota, Minneapolis, MN, United States; ^2^Department of Ophthalmology, New York University, New York, NY, United States; ^3^Department of Neurosurgery, University of California, San Diego, San Diego, CA, United States

**Keywords:** mast cells, neuroinflammation, neurodegenerative disease, Alzheimer’s, Parkinson’s, amyotrophic lateral sclerosis, Huntington’s

## Abstract

Neurodegenerative diseases affect millions of people worldwide, yet there are currently no effective treatments. Because risk of neurodegenerative disease substantially increases with age, greater life expectancy with a concomitant aging population means more individuals will be affected in the coming decades. Thus, there is an urgent need for understanding the mechanisms driving neurodegenerative diseases in order to develop improved treatment strategies. Inflammation in the nervous system, termed “neuroinflammation,” has become increasingly recognized as being associated with neurodegenerative diseases. Early attention focused primarily on morphological changes in astrocytes and microglia; however, brain and CNS resident mast cells are now receiving attention as a result of being “first responders” to injury. Mast cells also exert profound effects on their microenvironment and neighboring cells including behavior and/or activation of astrocytes, microglia, and neurons, which, in turn, are implicated in neuroinflammation, neurogenesis and neurodegeneration. Mast cells also affect disruption/permeability of the blood brain barrier enabling toxin and immune cell entry exacerbating an inflammatory microenvironment. Here, we discuss the roles of mast cells in neuroinflammation and neurodegeneration with a focus on development and progression of four prominent neurodegenerative diseases: Alzheimer’s Disease, Parkinson’s Disease, Amyotrophic Lateral Sclerosis, and Huntington’s Disease.

## Introduction

Mast cells are “first responders” that become activated with exposure to a diverse array of stimuli, from allergens and antigens to neuropeptides, trauma and drugs (Hendriksen et al., 2017). Activated mast cells are multifunctional effector cells that exert a variety of both immediate and delayed actions. Within minutes of stimulation, mast cells release granules containing preformed cytokines, biogenic amines, proteoglycans, proteases, leukotrienes, and lysosomal enzymes. Subsequent *de novo* synthesis and release of lipid mediators (e.g., leukotrienes, growth factors, prostaglandins) as well as cytokines and chemokines may sustain or oppose the early effects (Gupta and Harvima, 2018). Mast cells may also release extracellular vesicles, extracellular traps, and form nanotubes (Weng et al., 2016) that enable interactions with neighboring cells and structures including vessels and nerve fibers (Gupta and Harvima, 2018).

Myeloid progenitor cells from the bone marrow form immature mast cell precursors that migrate through the bloodstream to different tissues, where they undergo differentiation into mature mast cells and persist for long periods (Gupta and Harvima, 2018). Signals from the surrounding microenvironment and any attendant pathological conditions critically influence local mast cell size, structure, secretagog, sensitivity to stimuli and response to inhibitory signals/drugs. Mast cells may thus display substantial phenotypic heterogeneity between and within different organs including the nervous system (Metcalfe et al., 1997).

Chronic and acute inflammation in the nervous system, termed “neuroinflammation,” have been associated with several neurodegenerative diseases, including those discussed in this review. Acute and chronic inflammation are also involved in neuropathic pain (Gupta and Harvima, 2018). Hence, although its close proximity to, and extensive communication with, the immune system provides the nervous system with substantial protection, this same relationship also makes the nervous system highly vulnerable to severe pathologies that significantly impact quality of life. The role of mast cells in neurodegenerative diseases is being increasingly recognized. In this review, we present an overview of mast cell function within the central and peripheral nervous systems with specific attention to neuroinflammation and neurodegeneration. We then focus on the roles of mast cells in the development and progression of four prominent and devastating neurodegenerative diseases: Alzheimer’s Disease, Parkinson’s Disease, Amyotrophic Lateral Sclerosis and Huntington’s Disease.

## Mast Cell Localization in the Central and Peripheral Nervous Systems

Mast cells populate the brain during both development (Skaper et al., 2014) and adulthood, when they may migrate from the periphery to the brain (Nautiyal et al., 2011). The healthy human brain contains small numbers of mast cells located primarily in the abluminal perivascular areas and meninges (Banuelos-Cabrera et al., 2014; Dong et al., 2014), whereas mice have higher numbers of mast cells populating diverse regions of the brain (Nautiyal et al., 2012). Mast cells have been identified in the area postrema of the dorsal medulla, choroid plexus, and parenchyma of the thalami and hypothalamus (Ribatti, 2015; Hendriksen et al., 2017). The number and distribution of mast cells in the brain may change during infection, trauma, or stress (Bugajski et al., 1994; Maslinska et al., 2005; Silver and Curley, 2013).

Mast cells are also present the dura of the spinal cord, but not in the cord parenchyma under normal conditions. Nonetheless, mast cell mediators may still be able to modulate synaptic transmission and nociception at the level of the dorsal horn due to the close apposition of dura and white matter in this compartment (Michaloudi et al., 2008; Xanthos et al., 2011). Mast cells are also found in close proximity to peripheral nerves in tissues throughout the body (Schemann and Camilleri, 2013; Kritas et al., 2014a; Forsythe, 2015; Gupta and Harvima, 2018).

## Mast Cell Activation, Neuroinflammation, and Neurodegeneration

Hendriksen et al. (2017) have suggested a framework for characterizing the role of mast cells in neuroinflammation:

(1) Reciprocal interactions with microglia, astrocytes and neurons (Skaper et al., 2014)
(2) Effects on blood-brain barrier permeability (Hendriksen et al., 2017)
(3) Effects on neurogenesis: proliferation, differentiation, and migration (Molina-Hernandez and Velasco, 2008; Borsini et al., 2015)
(4) Effects on neurodegeneration: neuronal death, synaptic dysfunction, excitotoxicity (Kempuraj et al., 2017b)

A full discussion of any/all of these phenomena is beyond the scope of this review. Selected processes most relevant to neurodegenerative diseases are described below.

### Mast Cell-Microglia Interactions

In the brain and CNS, microglial cells are the guardian immune surveillance effectors that constantly monitor the surrounding microenvironment for injury and pathogen entry, which elicit microglial activation encompassing the release of cytokines/chemokines, phagocytosis of cellular debris and antigen presentation to T cells (Colonna and Butovsky, 2017). Cross-talk between microglial cells and other cells of the immune system enable complex, multifaceted communication between the brain, CNS and “first responders” that affords neural protection. Nevertheless, such homeostatic and protective responses are prone to dysregulation, particularly as a consequence of aging, which gives rise to chronic inflammation, resulting in tissue damage with a concomitant impaired ability to heal (Di Benedetto et al., 2017).

While microglial cells provide immune surveillance to the brain and CNS, other immune cells have recently been recognized for their contributions to neuronal degenerative diseases. Notably, tissue-resident mast cells have garnered much attention as primary communicators and mediators between the peripheral immune system and the nervous system during inflammatory responses (Gupta and Harvima, 2018). Mast cells have long been recognized for their roles in allergic inflammation and anaphylaxis; however, their localization within the CNS has led to recent exploration into their possible roles in neuroinflammation and neurodegenerative disease (Hendriksen et al., 2017). In fact, mast cells within the CNS, as opposed to microglial cells, are now recognized as the primary first responders to injury as conferred by their secretory granule arsenal of preformed/stored immunomodulators, neuromodulators, proteases, amines and growth factors (Gupta and Harvima, 2018). Mast cell progenitors are able to traverse the blood-brain-barrier (BBB) and blood-spinal cord-barrier under states of inflammation and infection (Nautiyal et al., 2011). In response to localized microenvironment perturbation, mast cells undergo activation in which pre- and newly synthesized mediators such as GnRH, monoamines, specific proteases (e.g., chymases, tryptases, and carboxypeptidase A), cytokines, and histamine are secreted by the process of degranulation (Metcalfe et al., 1997; Vukman et al., 2017). Release of these compounds elicits profound effects on neighboring cells including T cells, which are able to enter the brain via compromised BBB. Microglial cells are also activated in response to mast cell release of tryptase and histamine, resulting in a pro-inflammatory state mediated by microglial secretion of cytokines/chemokines into the microenvironment (Hendriksen et al., 2017). In addition, mast cells release chemoattractants, which recruit eosinophils, monocytes, and neutrophils further exacerbating an inflammatory environment (Jolly et al., 2004).

Interactions between mast and microglial cells involves complex cross-communication that can be both unidirectional and bidirectional. Activated microglia release IL-6 and chemokine (C-C motif) ligand 5 (CCL5), which affect surface expression levels of toll-like receptors, TLR2 and TLR4, on mast cells thus modulating the ability of mast cells to respond to endotoxins (Pietrzak et al., 2011). Conversely, release of CCL5/RANTES (regulated on activation, normal T cell expressed and secreted) by mast cells induces proinflammatory responses in microglial cells (Skuljec et al., 2011). Tryptase released from mast cells cleaves and activates protease activated receptor 2 (PAR2) on microglial cells resulting in the upregulation and release of brain-derived neurotrophic factor (BDNF); while IL-6 and TNF-α released from microglial cells upregulates PAR2 expression on mast cells, resulting in mast cell activation (Zhang and Levy, 2008; Zhang et al., 2010). C-X-C chemokine receptor type 4 (CXCR4) expression promotes migration and activation of microglial cells and also acts as a mast cell chemotaxin (Juremalm et al., 2000; Wang et al., 2008). ATP stimulates IL-33 release from microglial cells, which in turn induces IL-6, IL-13, and monocyte chemoattractant protein 1 secretion from mast cells, resulting in modulatory responses in microglial cells (Osipchuk and Cahalan, 1992; Bulanova and Bulfone-Paus, 2010; Taracanova et al., 2017). From these few examples, it is clear that mast cell-microglia interactions encompass highly complex paracrine mechanisms by which these cells, as a result of close proximity, influence each other’s behavior and responses to their microenvironment.

### Mast Cell Effects on Blood-Brain Barrier Permeability

The blood-brain barrier (BBB) plays a critical role in controlling the entry of molecules, pathogens, and toxins into the CNS. The primary barrier units of the BBB are tight junctions between endothelial cells (ECs) that limit paracellular transport. Tight junctions are composed of transmembrane proteins such as claudin and occludin (Strbian et al., 2009). BBB-specific receptors on ECs modulate trafficking of molecules into and out of the brain (Keaney and Campbell, 2015). Surrounding pericytes, astroglia and neurons communicate with ECs and impart further integrity and complexity to the BBB (Banuelos-Cabrera et al., 2014; Ribatti, 2015). Disruption and breakdown of the BBB is associated with a variety of neoplastic, infectious, inflammatory, and neurodegenerative diseases (Daneman and Prat, 2015; Ribatti, 2015). Mast cells are believed to influence BBB integrity through release of proteases that can degrade tight junction proteins and extracellular matrix components, as well as release of vasoactive mediators including histamine and TNF-α (Strbian et al., 2009; Mattila et al., 2011).

### Mast Cell Effects on Neurodegeneration

As a result of their ability to quickly release diverse immuno- and neuromodulators, the relatively small numbers of mast cells in the brain and CNS have a substantial influence on the behaviors of neurons and glial cells. Direct influences via release of TNF-α, histamine and proteases empower mast cells with the ability to potentiate neuroinflammation, neurogenesis, neurodegeneration and BBB disruption/permeability. Nevertheless, to date all evidence regarding the direct influence of mast cells on neurodegeneration derive from animal studies (Secor et al., 2000). It therefore remains to be determined the extent to which such findings hold true human relevance. With respect to neurodegeneration, mast cell release of TNF-α and other cytokines can increase neuroinflammation and the formation of neurotoxic nitric oxide by astrocytes. Cytokines such as IL-6, IL-1β, and TNF-α can elicit protective as well as detrimental effects and these differential outcomes are likely dependent on the concentration and duration with which these cytokines are expressed and released. TNF-α and IL-6 can affect the expression and function of tight junction proteins; and, therefore, the release of these cytokines is likely involved in the capacity of mast cells to modulate BBB permeability and the entry of immune cells and other molecules that do not have access under physiological conditions. The potential roles of histamine in neurogenesis and neurodegeneration have remained somewhat controversial since conflicting reports indicate that histamine is both neuroprotective and can also increase neurotoxicity (Skaper et al., 2001; Fang et al., 2014). Moreover, reports indicate that histamine can potentiate both increased BBB integrity and increased BBB permeability. It is likely that different histamine receptors may have diverse effects, and their presentation may thus guide context-specific histamine activity. The roles of mast cells in neurogenesis and neurodegeneration thus involve a high level of nuanced complexity.

## Mast Cells in Alzheimer’s Disease

Alzheimer’s Disease (AD) is the most common cause of dementia among the elderly population, with 5–7 million new diagnoses annually (Robinson et al., 2017). Patients experience progressive, disabling cognitive deficits and decline in learning and memory (Bianchetti et al., 2006). Although the cardinal histopathological features of extracellular amyloid β-peptide (Aβ) deposition, intracellular neurofibrillary tangles composed of hyperphosphorylated tau protein and synaptic loss have been extensively described, the exact etiology of AD remains unclear (Zlomuzica et al., 2016). The causative role of amyloid toxicity in AD pathogenesis has long been questioned (Yankner, 1989). Contemporary understanding suggests that Aβ oligomers may impair synaptic function and plasticity (Selkoe, 2008), with attendant derangement of multiple neurotransmitter systems and neuronal networks (Zlomuzica et al., 2016).

**FIGURE 1:**
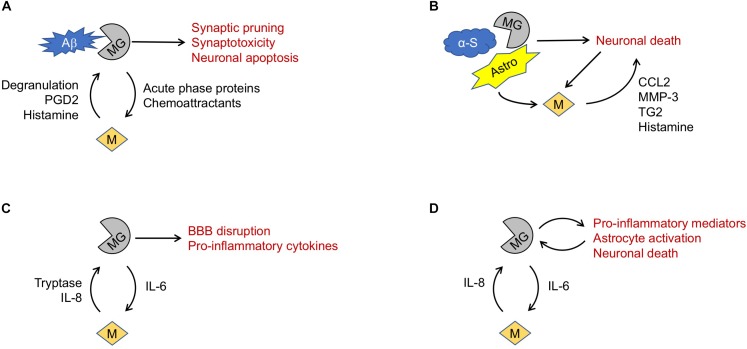
Proposed mechanisms of mast cell involvement in neurodegenerative diseases. **(A)** In Alzheimer’s Disease, microglia (MG) phagocytosis of amyloid β (Aβ) fragments may trigger release of mediators causing mast cell (M) degranulation. Mast cell products may in turn promote microglia-mediated neurotoxicity. **(B)** In Parkinson’s Disease, misfolded α-synuclein (α-S) may trigger microglia-mediated death of dopaminergic neurons. Astrocytes (Astro), microglia and dying neurons may all promote mast cell recruitment and release of mediators that exacerbate neuronal death. **(C)** In amyotrophic lateral sclerosis, TNF-α and IL-6 elaborated by microglia may drive mast cell recruitment, activation, and degranulation. Release of mediators such as tryptase and IL-8 can reciprocally activate microglia, exacerbating blood-brain barrier (BBB) disruption and release of pro-inflammatory cytokines. **(D)** Similarly, in Huntington’s Disease a feed-forward interaction between microglia and mast cells may promote a pro-inflammatory and neurotoxic milieu.

Recent studies increasingly implicate neuroinflammation in AD neurodegeneration (Heneka et al., 2015). For example, microglia have been found to surround Aβ plaques in postmortem AD brain specimens as well as mouse models of amyloid deposition (Skaper et al., 2018). Phagocytosed Aβ peptides have been shown to regulate microglia phenotype (Manocha et al., 2016), induce production of trophic factors (Rivest, 2009) and synaptotoxic compounds (Skaper et al., 2018), and trigger early widespread synaptic pruning (Hong et al., 2016) (Figure 1).

Autopsy studies have shown mast cells surround amyloid plaques in Alzheimer’s patients in higher numbers than corresponding brain regions of control patients (Maslinska et al., 2007). It has been suggested that inflammation in the AD brain may trigger CNS glia to produce acute phase proteins and mast cell chemoattractants such as serum amyloid A that home mast cells to sites of amyloid deposition (Skaper et al., 2018). It is also possible that mast cells themselves are early detectors of amyloid peptides; indeed, Harcha et al. (2015) showed increased mast cell numbers in the cortex and hippocampus of a mouse model of AD prior to amyloid detection. The authors also demonstrated that amyloid peptides can activate membrane Panx1 hemichannels on mast cells, leading to degranulation. Importantly, the authors demonstrated attenuation of these responses by Panx1 inhibitors *in vitro* as well as in the mast cells of *Panx1-/-* mice (Harcha et al., 2015). Subsequent release of mediators including histamine and prostaglandin D2 (PGD2) may exacerbate local inflammatory processes including microglial activation (Shaik-Dasthagirisaheb and Conti, 2016).

The oral tyrosine kinase inhibitor masitinib modulates mast cell degranulation, differentiation and survival through c-kit and Lyn targeting (Dubreuil et al., 2009). A phase 2 randomized, placebo-controlled trial of masitinib as add-on therapy for patients with mild-to-moderate AD showed reduction in the rate of cognitive decline over a 24-week period. The ability of mast cells to disrupt integrity of the BBB has been implicated in the stress-induced neuropathological processes involved in the development and progression of AD (Esposito et al., 2001; Mravec et al., 2018). Because masitinib is unlikely to cross the BBB, this was potentially achieved by inhibiting mast cells in close proximity to the BBB from releasing mediators that would impair BBB permeability, leading to a decrease in local proinflammatory molecules and further migration of mast cells into the brain (Piette et al., 2011). Masitinib is currently in further phase 2 and 3 clinical trials (Folch et al., 2016).

## Mast Cells in Parkinson’s Disease

Parkinson’s Disease (PD) is characterized by progressive motor deficits including rigidity, bradykinesia and resting tremor, alongside non-motor deficits that may evolve later in the disease course (Shulman et al., 2011). Recent studies have linked neuroinflammation, particularly microglial activation, to PD pathogenesis (Stojkovska et al., 2015). Misfolded α-synuclein can activate microglia via several receptors including major histocompatibility complex II (Harms et al., 2013) and signaling cascades involving NF-kB and MAPKs. These events promote migration, phagocytosis and lymphocyte recruitment by microglia, and trigger increased expression of mediators including TNF-α, IL-6, and cyclooxygenase-2 (COX-2), ultimately exacerbating death of dopaminergic neurons (Zhang et al., 2017).

Parkinson’s Disease pathogenesis is also thought to involve down-regulation of nuclear receptor Nurr1 in microglia and astrocytes, leading to increased production of mediators such as chemokine CCL2 that may promote apoptosis of dopaminergic neurons (Liu et al., 2017). BBB dysfunction has also been shown in animal models and validated in PD patients (Gray and Woulfe, 2015). In a PD mouse model with impaired BBB function, matrix metalloproteinase-3 (MMP-3) was shown to play a critical role in death of nigrostriatal dopaminergic neurons (Chung et al., 2013). In spite of the known effects of mast cells on BBB permeability and disruption, there is currently little direct evidence for a role of mast cells in the pathogenesis of PD. Nevertheless, initial presumptive findings suggest that mast cells may contribute to PD via neural cell mediated activation. Kempuraj et al. (2016) demonstrated that exposure to dopaminergic toxin triggers release of CCL2 and MMP-3 by human umbilical cord blood-derived cultured mast cells and mouse bone marrow-derived mast cells (BMMCs) *in vitro*. The authors further characterized this phenomenon by exposing mast cells to glial activating factors in co-culture with fetal mouse brain-derived astrocytes, neurons, and/or mixed glia/neurons. Release of specific inflammatory mediators and neurite outgrowth were both quantified. By comparing all possible combinations of cell types, the authors demonstrated that mast cells specifically release mediators including tryptase. Furthermore, mast cell-specific mediators were shown to trigger CCL2 and MMP-3 release by astrocytes and glia in this model system. Taken together, these results suggest mast cell interactions with neurons and glial cells may play a role in PD pathogenesis (Kempuraj et al., 2018).

Hong et al. (2018) recently demonstrated that CCL2 production by microglia and astrocytes may recruit mast cells into the substantia nigra in a PD mouse model. Recruited mast cells were shown to express the cross-linking enzyme tissue transglutaminase 2 (TG2) in an NF-kB dependent manner, with subsequent release of pro-inflammatory mediators including histamine, leukotrienes, and TNF-α that are implicated in dopaminergic neuronal death. Although the comparison against TG2 knockout mice supported these findings, validation was not carried out in mast cell knockout controls. The authors also found increased TG2 expression in the serum of PD patients compared to control patients (Hong et al., 2018).

These results suggest intriguing roles for mast cells in PD pathogenesis, but should be interpreted cautiously when considering disease in humans. Animal and *in vitro* model systems may not fully recapitulate complex local microenvironments that substantially alter mast cell phenotype. Furthermore, postmortem studies of PD brain specimens have not consistently shown increased numbers of mast cells by conventional detection methods (Hurley et al., 2015).

## Mast Cells in Amyotrophic Lateral Sclerosis

Amyotrophic lateral sclerosis (ALS) involves progressive degeneration of both upper and lower motor neurons, often accompanied by cognitive and/or behavioral symptoms. ALS is the most common and aggressive form of motor neuron degeneration in adults, with a heterogenous but invariably progressive and fatal disease course (Hardiman et al., 2017). The underlying etiology remains unknown. Although some patients have inherited familial disease, the majority of cases are sporadic. The common pathologic feature is accumulation of ubiquitylated cytoplasmic protein inclusions in motor neurons. These inclusions are composed of transactive response (TAR) DNA binding protein 43 (TDP43) aggregates in the majority of cases (Neumann et al., 2006). Rodent models of ALS predominantly overexpress superoxide dismutase 1 (SOD1), which has also been implicated in some human cases (Hardiman et al., 2017).

Microglial activation has been shown in *SOD1*-transgenic mice (Liao et al., 2012) as well as human postmortem brain specimens and *in vivo* imaging in ALS patients (Turner et al., 2004; Corcia et al., 2012; Brites and Vaz, 2014). Accumulation of degranulating mast cells associated with macrophages at the neuromuscular junction has been shown to occur after onset of motor weakness in a rat model of ALS and correlate with denervation. Although a mast cell knockout control was not employed, treatment with the mast cell inhibitor masitinib reduced mast cell numbers and progression of motor symptoms, suggesting a cell type-specific effect (Trias et al., 2017). The mast cell chemoattractant IL-15 is elevated in the serum and cerebrospinal fluid of ALS patients (Rentzos et al., 2010), and mast cells expressing IL-17 have been found in the spinal cord of ALS patients (Fiala et al., 2010).

Cytokines including IL-12 are elevated in the serum and cerebrospinal fluid (CSF) of ALS patients (Rentzos et al., 2010). High mobility group box 1 protein and other damage-associated host biomolecules that can trigger an inflammatory response through TLR2/TLR4 signaling are elevated in the spinal cords of ALS patients (Casula et al., 2011). Yang et al. (2010) demonstrated that IL-12 can upregulate mast cell expression of TLR2/TLR4 pattern recognition receptors; although this was carried out in a mouse mastocytoma cell line *in vitro*, the findings support a possible role for mast cell autocrine signaling in the neuroinflammatory cascade.

Elevated IL-6 and IL-8 levels were found in the peripheral blood of ALS patients (Ehrhart et al., 2015). TNF-α and IL-6 elaborated by microglia have been shown to drive mast cell recruitment, activation, and degranulation, releasing mediators such as tryptase, which can reciprocally activate microglia in a feed-forward cycle in a rat model of PD (Zhang et al., 2010, 2011; Skaper et al., 2018). Mast cell tryptase has also been shown to stimulate microglial protease-activated receptors (PARs) that can cause disruption of the BBB in wild-type as compared to PAR-deficient mice (Bunnett, 2006). Human mast cell lines have been observed to release IL-8 (Chen et al., 2016; Yu et al., 2016). IL-8 enhances production of pro-inflammatory cytokines by microglia exposed to Aβ peptide *in vitro* (Franciosi et al., 2005). Taken together, these findings suggest further involvement of mast cells in cross-talk with microglia in the neuroinflammatory milieu of ALS.

Human and animal studies have also demonstrated impairment of the BBB and blood-spinal cord barriers in ALS (Rodrigues et al., 2012). As a result of pre-synthesized vasoactive mediators, mast cells affect the permeability and integrity of both the BBB (Ribatti, 2015) and blood-spinal cord-barrier. Therefore, in the case of ALS, mast cells have the potential to cross the blood-spinal cord-barrier and release neuropeptides, proteases, cytokines, histamine etc., via degranulation resulting in localized neuroinflammation and dysregulated neuronal function. Support for this, as stated above, comes from the finding that mast cells expressing IL-17 were present in the spinal cords of ALS patients. Further studies are needed to demonstrate and characterize the degree to which mast cells may be involved in these processes via the inflammatory and vasoactive mediators described above. Studies in animal models of ALS will be critical for validating the findings from different disease and *in vitro* contexts.

## Mast Cells in Huntington’s Disease

Huntington’s Disease (HD) is an autosomal dominant disorder caused by expansion of a CAG triplet in the *HTT* gene that leads to expression of a mutant form of the Huntington protein, HTT. Mutant HTT (mHTT) causes excitatory neurotoxicity in inhibitory medium spiny neurons (MSNs) in the striatum and cortex. Patients experience involuntary jerking movements as well as dystonia, rigidity, cognitive, and neuropsychiatric symptoms (Zuccato and Cattaneo, 2014).

Although the role of mast cells in HD pathogenesis has not yet been established, substantial evidence supports a strong role of neuroinflammation via interactions between neurons, microglia and astrocytes. Expression of mHTT in astrocytes downregulates production of neuronal growth factors, while mHTT expression in microglia promotes expression of proinflammatory cytokines (IL-6, TNF-α) and toxic metabolites. These processes converge to cause neurodegeneration, exacerbated by mHTT expression in neurons that triggers cell-autonomous apoptosis and degeneration. Components of dead neurons may be detected and phagocytosed by microglia in a similar fashion to Aβ detection in AD, resulting in further production of proinflammatory mediators, astrocyte activation, and a feed-forward loop of neuronal damage (Crotti et al., 2014; Crotti and Glass, 2015).

Plasma levels of IL-6 and IL-8 correlate with functional scores in HD patients (Bouwens et al., 2017). Several signaling cascades were found to drive microglia overexpression of IL-8 in a porcine model of HD (Valekova et al., 2016). Similar to findings in ALS, it is possible that these represent previously unexplored evidence of mast cell involvement in HD pathogenesis, and will require rigorous validation in animal models of HD.

## Future Directions

Emerging studies implicate chronic stress in a variety of neuroinflammatory processes (Machado et al., 2014) that may increase the risk of developing neurodegenerative disease (Hoeijmakers et al., 2017; Piirainen et al., 2017). Mast cells release and respond to molecules such as corticotropin releasing hormone during stress and neuroinflammation, suggesting a role in the pathogenesis of stress-related neurodegeneration and neuroinflammation (Kritas et al., 2014b). However, these mechanisms have yet to be definitively elucidated in the setting of neurodegenerative disease (Kempuraj et al., 2017a; Skaper et al., 2018). Similarly, the immune modulatory and neuroprotective role of gut microbiota has also been hypothesized to involve mast cells (Girolamo et al., 2017), suggesting another fruitful avenue for integrative mechanistic studies.

There are additional challenges and opportunities for future studies that are beyond the scope of this review. Possible neuroprotective roles of mast cells warrant further investigation. The dependence of mast cell phenotype on tissue- and pathology-specific microenvironment necessitates careful selection of animal and *in vitro* model systems, as well as validation in human tissue specimens. Further studies are also needed to elucidate the multifaceted cross-talk between mast cells and microglia, astrocytes and neurons. The scale of this problem may require informatics-based approaches; for example large genomic datasets derived from experimental mouse models may enable *in silico* discovery of target genes and guide rational validation in appropriate model systems (Khayer et al., 2017).

## Conclusion

A complete understanding of the mechanisms driving the development of neurodegenerative diseases is lacking. This has impeded the advancement of effective therapeutic strategies aimed at preventing both disease onset and progression. Although the many neurodegenerative diseases thus far identified have diverse characteristics and etiologies, many contributing factors are likely shared in common, which offers the possibility of identifying novel targets for intervention. Neuroinflammation, which is now recognized as a primary pathological component of diseases such as multiple sclerosis, is gaining acceptance as an underlying component of most, if not all, neurodegenerative diseases. Whereas past focus has predominantly centered on glial cells of the CNS, recently mast cells have emerged as potential key players in both neuroinflammation and neurodegenerative diseases. Mast cells are well positioned for such a role owing to their ability to affect both their microenvironment and neighboring cells including T cells, astrocytes, microglia, and neurons. The secretory granules of mast cells contain an arsenal of preformed/stored immunomodulators, neuromodulators, proteases, amines and growth factors that enable complex cross-communication, which can be both unidirectional and bidirectional. Mast cells can also affect disruption/permeabilization of the BBB and this has the potential for dramatically altering the neuroinflammatory state.

With respect to AD, PD, ALS, and HD, discussed in the present review, mast cell perturbation of the BBB appears to share a commonality. Moreover, mast cells have been found to home to sites of amyloid deposition in AD; and, an inhibitor of mast cell function was shown to reduce cognitive decline in AD patients. Mast cell interactions with neurons and glial cells have also been implicated in PD pathogenesis. Emerging evidence suggests that mast cell autocrine signaling may contribute to ALS: The mast cell chemoattractant, IL-15, is elevated in the serum and cerebrospinal fluid of ALS patients; and, mast cells expressing IL-17 have been found in the spinal cord of ALS patients. Plasma levels of cytokines (IL-6, IL-8), known to affect mast cell activation, have been correlated with functional scores in HD patients suggesting the possible involvement of mast cells in the pathogenesis of HD. Future considerations include validation of animal and *in vitro* models, which incorporates microenvironment-specific influences and the complex, multifaceted cross-talk between mast cells and microglia, astrocytes and neurons. In addition to the potential role(s) of mast cells in neuroinflammation and neurodegenerative diseases, the possible neuroprotective roles of mast cells also warrant further investigation.

## Author Contributions

All authors participated in the conceptualization, literature review, drafting, and editing of the manuscript.

## Conflict of Interest Statement

The authors declare that the research was conducted in the absence of any commercial or financial relationships that could be construed as a potential conflict of interest.
